# Dynamics of COVID-19 mathematical model with stochastic perturbation

**DOI:** 10.1186/s13662-020-02909-1

**Published:** 2020-08-28

**Authors:** Zizhen Zhang, Anwar Zeb, Sultan Hussain, Ebraheem Alzahrani

**Affiliations:** 1 School of Management Science and Engineering, Anhui University of Finance and Economics, Bengbu, 233030 China; 2 Department of Mathematics, COMSATS University Islamabad, Abbottabad Campus, Abbottabad, 22060 Khyber Pakhtunkhwa Pakistan; 3 Department of Mathematics, Faculty of Science, King Abdulaziz University, P. O. Box 80203, Jeddah, 21589 Saudi Arabia

**Keywords:** Stochastic COVID-19 model, Itô’s formula, Extinction, Persistence, Numerical analysis

## Abstract

Acknowledging many effects on humans, which are ignored in deterministic models for COVID-19, in this paper, we consider stochastic mathematical model for COVID-19. Firstly, the formulation of a stochastic susceptible–infected–recovered model is presented. Secondly, we devote with full strength our concentrated attention to sufficient conditions for extinction and persistence. Thirdly, we examine the threshold of the proposed stochastic COVID-19 model, when noise is small or large. Finally, we show the numerical simulations graphically using MATLAB.

## Introduction

There are many people who are currently alert of the outburst of COVID-19, which was recognized in China in December of 2019. As of this conformation, each continent has been influenced by this profoundly infectious disease, with about million cases analyzed in more than 200 nations around the world. The reason for this episode is another infection, known as the extremely intense respiratory disorder coronavirus 2 (SARS-CoV-2). On February 12, 2020, WHO named this disease coronavirus. The rapid spread of coronavirus COVID-19 is of great interest and has the attention of governments, medical doctors and public/private health organizations because of its high rate of spreading and the significant number of deaths that occurred specially in China, Italy, Iran, USA, UK, Turkey, Pakistan, and India. In the meantime, many doctors, mathematicians, pharmacists, biologists and chemists are trying to study the behavior of COVID-19, which is a pandemic initiated from China [1]. Actually, this virus was initiated from Wuhan, China. This is a vector transmission because its required source is in the form of human-to-human spread. It means the vector for this disease is people; so far all the governments restricted the people to keep distance from each other but the public is careless in this situation. On the mathematical side, the authors applied modified SIR (susceptible, infected and recovered), SEIR (susceptible, exposed, infected and recovered) and SIRS (susceptible, infected and recovered, susceptible) models to determine the actual number of infected by COVID-19, and specific burdens on isolation wards and intensive care units, similarly, using different scenarios for how to control the quick spread of this viral disease. Nesteruk [2], studied the SIR model for control of this pandemic. But there is no one until now who could control this virus. If we make the contact rates very small it will show the best effect on the further spreading of COVID-19, so for this purpose all governments take action for in terms of the household effect. For the estimation of the final size of the coronavirus epidemic, Batista [3] presented the logistic growth regression model. Many researchers discussed this COVID-19 in different models in integer and in fractional order, see [1–17], because of many applications of fractional calculus, stochastic modeling and bifurcation analysis [18–26]. For the more realistic models, several authors studied the stochastic models by introducing white noise [27–31]. The effects of the environment in the AIDS model were studied by Dalal et al. [27] using the method of parameter perturbation. Stochastic models will likely produce results different from deterministic models every time the model is run for the same parameters. Stochastic models possess some inherent randomness. The same set of parameter values and initial conditions for deterministic models will lead to an ensemble of different outputs. Tornatore et al. [28–30] studied the stochastic epidemic models with vaccination. In this work, they proved the existence, uniqueness, and positivity of the solution. A stochastic SIS epidemic model containing vaccination is discussed by Zhu et al. [31]. They obtained the condition of the disease extinction and persistence according to noise and threshold of the deterministic system. Similarly, several authors discussed the same conditions for stochastic models; see [32–39].

To study the effects of the environment on spreading of COVID-19 and make the research more realistic, first we formulate a stochastic mathematical COVID-19 model. Then sufficient conditions for extinction and persistence are examined. Furthermore, the threshold of the proposed stochastic COVID-19 model is determined. It plays an important role in mathematical models as a backbone, when there is small or large noise. Finally, we show the numerical simulations graphically with the aid of MATLAB.

The rest of the paper is organized as follows: Sect. 2 is concerned with the COVID-19 model with random perturbation formulation. Section 3 is related to the unique positive solution of proposed model. Furthermore, we investigate the exponential stability of the proposed model in Sect. 4. The persistent conditions are shown in Sect. 5. Finally, we conclude with the results and outcomes of the paper in Sect. 6.

## Model formulation

In this section, a COVID-19 mathematical model with random perturbation is formulated as follows:

1 $$\begin{aligned} \begin{gathered}\frac{dS(t)}{dt} =\varLambda-\beta S(t)I(t)-\mu S(t)+ \delta R(t)-\rho S(t)I(t)\,dB(t) , \\ \frac{dI(t)}{dt} =\beta S(t)I(t)-(\gamma+\mu)I+\rho S(t)I(t)\,dB(t), \\ \frac{dR(t)}{dt} =\gamma I(t)-\mu R(t)-\delta R(t), \end{gathered} \end{aligned}$$

where the description of parameters and variables are given in Table 1.

**Table 1:** Parameters and description

Notations	Description
*S*(*t*)	Represents susceptible population
*I*(*t*)	Represents infectious people class
*R*(*t*)	Denotes recovered population
*Λ*	The joining rate of people to susceptible class through birth or migration
*β*	Rate at which the susceptible tends to infected class
*μ*	Represents natural and due to coronavirus death
*γ*	Represents the recovered rate
*B*(*t*)	The standard Brownian motions, with $\rho^{2}>0$ and with intensity of white noise
*δ*	The rate of deteriorate in health

In deterministic form the model (1) is given by

2 $$\begin{aligned}& \frac{dS(t)}{dt} =\varLambda-\beta S(t)I(t)-\mu S(t)+ \delta R(t) , \\& \frac{dI(t)}{dt} =\beta S(t)I(t)-(\gamma+\mu)S, \\& \frac{dR(t)}{dt} =\gamma I(t)-\mu R(t)-\delta R(t), \end{aligned}$$

and

3 $$ \frac{dN}{dt}=\varLambda-\mu N, $$

where $N(t)=S(t)+I(t)+R(t)$ shows the total constant population for $\varLambda\approx\mu N$ and $N(0)=S(0)+I(0)+R(0)$. Equation (3) has the exact solution

4 $$ N(t)=e^{-\mu t} \biggl[N(0)+\frac{\varLambda}{\mu}e^{\mu t} \biggr]. $$

Also, we have

$$ S(0)\geq0,~I(0)\geq0,~R(0)\geq0\quad \Longrightarrow \quad S(t)\geq0,~I(t)\geq 0,~R(t) \geq0. $$

So, the solution has a positivity property. For stability analysis of model (2), we have the reproductive number, which is

5 $$ R_{0}=\frac{\beta}{\gamma+\mu}N. $$

If $R_{0}<1$, then system (2) will be locally stable and unstable if $R_{0}\geq1$. Similarly for $\Lambda=0$, the system (2) will be globally asymptotically stable.

## Existence and uniqueness of the positive solution

Here, we first make the following assumptions:

- Set $R_{+}^{d}=\{\chi_{i}\in R^{d},\chi_{i}>0,1\leq d\}$.
- Suppose a complete probability space $(\varOmega,\mathfrak {F},\{\mathfrak{F}\}_{t\geq0},P )$ with filtration $\{\mathfrak {F}\}_{t\geq0}$, which satisfies the usual conditions.

Generally, consider a stochastic differential equation of *n*-dimensions as

6 $$\begin{aligned} dx(t) & =F\bigl(y(t),t\bigr)\,dt+G\bigl(y(t),t\bigr)\,dB(t),\quad \text{for } t\geq t_{0}, \end{aligned}$$

with initial value $y(t_{0})=y_{0}\in R^{d}$. By defining the differential operator *L* with Eq. (6)

7 $$\begin{aligned} L & =\frac{\partial}{\partial t}+\sum^{d}_{i=1}F_{i}(y,t) \frac{\partial}{\partial y_{i}}+\frac{1}{2}\sum^{d}_{i,j=1}{ \bigl[}G^{T}(y,t)G(y,t){ \bigr]}_{ij} \frac{\partial^{2}}{\partial y_{i}\,\partial y_{j}}. \end{aligned}$$

If the operator *L* acts on a function $V=(\mathbb{R}^{d}\times\tilde{\mathbb{R}}_{+};\tilde{\mathbb{R}}_{+})$, then

8 $$\begin{aligned} L V(y,t) & =V_{t}(y,t)+V_{y}(y,t)F(y,t)+ \frac{1}{2}\operatorname{trace}{ \bigl[}G^{T}(y,t)V_{yy}(y,t)G(y,t){ \bigr]}. \end{aligned}$$

### Theorem 3.1

*There is a unique positive solution* $(S(t), I(t),R(t) )$*of system* (1) *for* $t\geq0$*with* $(S(0), I(0),R(0) )\in R_{+}^{3}$, *and solution will be left in* $R_{+}^{3}$, *with probability* 1.

### Proof

Since the coefficient of the differential equations of system (1) are locally Lipschitz continuous for $(S(0), I(0),R(0) )\in R_{+}^{3}$, there is a unique local solution $(S(t), I(t),R(t) )$ on $t\in[0,\tau_{e})$, where $\tau_{e}$ is the time for noise caused by an explosion (see [6]). For demonstrating the solution to be global, it is sufficient that $\tau_{e}=\infty$ a.s. Suppose that $k_{0}\geq0$ is sufficiently large so that $(S(0), I(0),R(0) )\in[\frac{1}{k_{0}},k_{0}]$. For each integer $k\geq k_{0}$, define the stopping time

$$\tau_{e}=\inf \biggl[t\in[0,\tau_{e}):\min \bigl(S(t), I(t),R(t) \bigr)\leq\frac{1}{k_{0}}\text{ or }\max \bigl(S(t), I(t),r(t) \bigr)\geq k \biggr], $$

where we set $\inf \phi(\text{empty set})=\infty$ throughout the paper. For $k\rightarrow\infty$, $\tau_{k}$ is clearly increasing. Set $\tau_{\infty}=\lim_{k\rightarrow\infty}\tau_{k}$ whither $\tau_{\infty}\leq\tau_{e}$. If we can show that $\tau_{\infty}=\infty$ a.s, then $\tau_{e}=\infty$. If false, then there are a pair of constants $T>0$ and $\epsilon\in(0,1)$ such that

$$P\{\tau_{\infty}\leq T\}>\epsilon. $$

So there is an integer $k_{1}\geq k_{0}$, which satisfies

$$P\{\tau_{k}\leq T\}\geq\epsilon \quad\text{for all }k\geq k_{1}. $$

Define a $C^{2}$-function $V:\mathbb{R}^{3}_{+}\rightarrow\tilde{\mathbb{R}}_{+}$ by

9 $$\begin{aligned} V (S,I,R ) & ={ \biggl(}S-c-c\ln \frac{S}{c}{ \biggr)}+{ (}I-1-\ln I{ )}+{ (}R-1-\ln R{ )}. \end{aligned}$$

By applying the Itô formula, we obtain

10 $$\begin{aligned} dV (S,I,R ) & = \biggl(1-\frac{c}{S} \biggr)\,dS+\frac {1}{2S^{2}}(dS)^{2}+ \biggl(1-\frac{1}{I} \biggr)\,dI+\frac {1}{2I^{2}}(dI)^{2}+ \biggl(1-\frac{1}{R} \biggr)\,dR \end{aligned}$$

11 $$\begin{aligned} & = LV\,dt+\rho (I-S )\,dB(t), \end{aligned}$$

where $LV:\mathbb{R}^{3}_{+}\rightarrow\tilde{\mathbb{R}}_{+}$ is defined by

$$\begin{aligned} LV & = \biggl(1-\frac{c}{S(t)} \biggr) \bigl(\varLambda-\beta S(t)I(t)- \mu S(t)+\delta R(t) \bigr)+\frac{1}{2}\rho^{2}I^{2} \\ &\quad+ \biggl(1-\frac{1}{I} \biggr) \bigl(\beta S(t)I(t)-(\gamma+\mu )I \bigr)+\frac{1}{2}\rho^{2}S^{2}+ \biggl(1- \frac{1}{R} \biggr) \bigl(\gamma I(t)-\mu R(t)-\delta R(t) \bigr) \\ &=\varLambda-\beta S(t)I(t)-\mu S(t)+\delta R(t)-\frac{c\varLambda }{S(t)}+c\beta I(t)+c\mu-c\delta\frac{R(t)}{S(t)}+\frac{1}{2}\rho ^{2}I^{2} \\ &\quad+\beta S(t)I(t)-(\gamma+\mu)I(t)-\beta S(t)+(\gamma+\mu)+\frac {1}{2} \rho^{2}S^{2}+\gamma I(t)-\mu R(t)-\delta R(t) \\ &\quad-\gamma\frac{I(t)}{R(t)}+\mu+\delta \\ &\leq\varLambda-(\gamma+\mu)I+c\beta I(t)+c\mu+\gamma+\mu+\mu +\delta+ \frac{1}{2}\rho^{2}I^{2}+\frac{1}{2} \rho^{2}S^{2}. \end{aligned}$$

By choosing $c=\frac{\gamma+\mu}{\beta}$, it follows that

12 $$\begin{aligned} LV \leq\varLambda+c\mu+\gamma+\mu+\mu+\delta+\frac{1}{2}\rho ^{2}I^{2}+\frac{1}{2}\rho^{2}S^{2} \triangleq B. \end{aligned}$$

Further proof follows from Ji et al. [31]. □

## Extinction

In this section, we investigate the condition for extinction of the spread of the coronavirus. Here, we define

13 $$\begin{aligned} \bigl\langle y(t)\bigr\rangle =\frac{1}{t} \int_{0}^{t}y(s)\,ds \end{aligned}$$

and

14 $$\begin{aligned} \tilde{\Re}=\beta \biggl(\frac{\varLambda}{\mu} \biggr)\frac {1}{(\gamma+\mu)+\frac{1}{2}\rho^{2}(\frac{\varLambda}{\mu})^{2}}. \end{aligned}$$

A useful lemma concerned with this work is as follows.

### Lemma 4.1

([31])

*Let* $M=\{M_{t}\}_{t\geq0}$*have a real value*, *and be continuous*, *local martingale and vanishing at* $t=0$. *Then*

$$\lim_{t\rightarrow\infty}\langle M, M\rangle_{t}=\infty $$

*a*.*s*. *implies that*

$$\lim_{t\rightarrow\infty}\frac{M_{t}}{\langle M, M\rangle_{t}}=0 $$

*and also*

$$\lim_{t\rightarrow \infty}\sup\frac{\langle M, M\rangle_{t}}{t}< \infty\quad\Longrightarrow\quad \lim_{t\rightarrow\infty}\frac{M_{t}}{t}=0. $$

### Theorem 4.1

*Let* $(S(t),I(t),R(t))$*be the solution of system* (1) *with initial value* $(S(0),I(0), R(0))\in\in{R_{+}^{3}}$. *If*

1. $\rho^{2}>\max ({\frac{\beta^{2}}{2(\gamma+\delta+\mu +\alpha)},\frac{\beta\mu}{\varLambda}} )$, *or*
2. $\tilde{R}<1$*and* $\rho^{2}\leq\frac{\beta\mu}{\varLambda}$.

*Then*

15 $$\begin{aligned}& \lim_{t\rightarrow\infty}\sup\frac{\log I(t)}{t}\leq-( \gamma+\mu )+\frac{\beta}{2\rho^{2}}< 0\quad\textit{a.s. if }(1)\textit{ holds}, \end{aligned}$$

16 $$\begin{aligned}& \lim_{t\rightarrow\infty}\sup\frac{\log I(t)}{t}\leq\beta \frac {\varLambda}{\mu} \biggl(1-\frac{1}{\tilde{\Re}} \biggr)< 0\quad \textit{a.s. if } (2) \textit{ holds}. \end{aligned}$$

*In addition*

$$\lim_{t\rightarrow\infty}S(t)=\frac{\varLambda}{\mu}=S_{0},\quad\quad \lim_{t\rightarrow\infty}I(t)=0\quad \textit{and} \quad\lim_{t\rightarrow\infty }R(t)=0,\quad \textit{a.s.} $$

### Proof

Performing the integration of system (1)

$$\begin{aligned}& \frac{S(t)-S(0)}{t} = \varLambda-\beta\bigl\langle S(t)I(t)\bigr\rangle -\mu \bigl\langle S(t)\bigr\rangle +\delta\bigl\langle R(t)\bigr\rangle -\rho S(t)I(t)\,dB(t), \\& \frac{I(t)-I(0)}{t} = \beta\bigl\langle S(t)I(t)\bigr\rangle -( \gamma+\mu )\bigl\langle I(t)\bigr\rangle +\rho S(t)I(t)\,dB(t), \\& \frac{R(t)-R(0)}{t} = \gamma\bigl\langle I(t)\bigr\rangle -(\mu+\delta) \bigl\langle R(t)\bigr\rangle . \end{aligned}$$

Then we have

$$\begin{aligned} &\frac{S(t)-S(0)}{t}+\frac{I(t)-I(0)}{t}+\frac{\delta}{\mu+\delta } \frac{R(t)-R(0)}{t}\\ &\quad= \varLambda-\beta\bigl\langle S(t)I(t)\bigr\rangle -\mu \bigl\langle S(t)\bigr\rangle +\delta\bigl\langle R(t)\bigr\rangle -\rho S(t)I(t)\,dB(t) \\ &\qquad{}+ \beta\bigl\langle S(t)I(t)\bigr\rangle -(\gamma+\mu)\bigl\langle I(t)\bigr\rangle +\rho S(t)I(t)\,dB(t) \\ &\qquad{}+ \gamma\bigl\langle I(t)\bigr\rangle -(\mu+\delta) \bigl\langle R(t)\bigr\rangle \\ &\quad= \varLambda-\mu\bigl\langle S(t)\bigr\rangle - \biggl((\gamma+\mu)- \frac {\delta\gamma}{\mu+\delta} \biggr)\bigl\langle I(t)\bigr\rangle \\ &\quad= \varLambda-\mu\bigl\langle S(t)\bigr\rangle - \biggl(\frac{(\gamma+\mu )(\mu+\delta)-\gamma\delta}{ \mu+\delta} \biggr)\bigl\langle I(t)\bigr\rangle \\ &\bigl\langle S(t)\bigr\rangle =-\frac{1}{\mu} \biggl[ \frac {S(t)-S(0)}{t}+\frac{I(t)-I(0)}{t}+\frac{\delta}{\mu+\delta} \frac {R(t)-R(0)}{t} \biggr] \\ &\phantom{\bigl\langle S(t)\bigr\rangle =}{}+ \frac{\varLambda}{\mu}-\frac{1}{\mu} \biggl(\frac{(\gamma+\mu )(\mu+\delta)-\gamma\delta}{\mu+\delta} \biggr)\bigl\langle I(t)\bigr\rangle . \end{aligned}$$

By applying $\lim_{t\rightarrow0}$

17 $$\begin{aligned}& \bigl\langle S(t)\bigr\rangle =\frac{\varLambda}{\mu}- \frac{1}{\mu} \biggl(\frac{(\gamma+\mu)(\mu+\delta)-\gamma\delta}{\mu+\delta} \biggr)\bigl\langle I(t)\bigr\rangle , \end{aligned}$$

18 $$\begin{aligned}& d\log I(t)= \biggl(\beta S-(\gamma+\mu)- \frac{1}{2}\rho^{2}S^{2} \biggr)\,dt+\rho S\,dB(t), \end{aligned}$$

19 $$\begin{aligned}& \frac{\log I(t)-\log I(0)}{t}=\beta\bigl\langle S(t)\bigr\rangle -(\gamma +\mu)-\frac{1}{2}\rho^{2}\bigl\langle S(t)^{2}\bigr\rangle +\frac{\rho}{t} \int _{0}^{t}S(r)\,dB(r) \end{aligned}$$

20 $$\begin{aligned}& \phantom{\frac{\log I(t)-\log I(0)}{t}}{}\leq\beta\bigl\langle S(t)\bigr\rangle -(\gamma+\mu)-\frac{1}{2}\rho ^{2}\bigl\langle S(t)\bigr\rangle ^{2}+ \frac{\rho}{t} \int_{0}^{t}S(r)\,dB(r). \end{aligned}$$

By putting in the value of $\langle S(t)\rangle$ from Eq. (17)

$$\begin{aligned} \frac{\log I(t)-\log I(0)}{t} \leq&\beta \biggl[\frac{\varLambda }{\mu}- \frac{1}{\mu} \biggl(\frac{(\gamma+\mu)(\mu+\delta )-\gamma\delta}{\mu+\delta} \biggr)\bigl\langle I(t)\bigr\rangle \biggr]-(\gamma+\mu) \\ &{}-\frac{1}{2}\rho^{2} \biggl[\frac{\varLambda}{\mu}- \frac{1}{\mu } \biggl(\frac{(\gamma+\mu)(\mu+\delta)-\gamma\delta}{\mu+\delta } \biggr)\bigl\langle I(t)\bigr\rangle \biggr]^{2}+\frac{\rho}{t} \int _{0}^{t}S(r)\,dB(r) \\ =&\beta \biggl[\frac{\varLambda}{\mu}- \biggl(\frac{\gamma+\mu }{\mu+\delta} \biggr)\bigl\langle I(t)\bigr\rangle \biggr]-(\gamma+\mu) \\ &{}-\frac{1}{2}\rho^{2} \biggl[ \biggl( \frac{\varLambda}{\mu} \biggr)^{2}- \biggl(\frac{\gamma+\mu}{\mu+\delta} \biggr)^{2}\bigl\langle I(t)\bigr\rangle ^{2} \biggr]+2 \frac{\varLambda}{\mu} \biggl(\frac{\gamma +\mu}{\mu+\delta} \biggr)\bigl\langle I(t)\bigr\rangle \\ &{}+\frac{\rho}{t} \int _{0}^{t}S(r)\,dB(r) \\ =& \frac{\beta\varLambda}{\mu}-(\gamma+\mu)-\frac{1}{2}\rho ^{2} \biggl(\frac{\varLambda}{\mu} \biggr)^{2}- \biggl( \frac{\beta(\gamma +\mu)}{\mu+\delta} \biggr)\bigl\langle I(t)\bigr\rangle \\ &{}+2\frac{\varLambda}{\mu} \biggl(\frac{\gamma+\mu}{\mu+\delta } \biggr)\bigl\langle I(t) \bigr\rangle -\frac{1}{2}\rho^{2} \biggl[- \biggl( \frac {\gamma+\mu}{\mu+\delta} \biggr)^{2}\bigl\langle I(t)\bigr\rangle ^{2} \biggr] \\ &{}+\frac{\rho}{t} \int_{0}^{t}S(r)\,dB(r) \\ =& \frac{\beta\varLambda}{\mu}- \biggl[(\gamma+\mu)+\frac {1}{2} \rho^{2} \biggl(\frac{\varLambda}{\mu} \biggr)^{2} \biggr]- \biggl(\frac{\beta(\gamma+\mu)}{\mu+\delta} \biggr)\bigl\langle I(t)\bigr\rangle \\ &{}+2\frac{\varLambda}{\mu} \biggl(\frac{\gamma+\mu}{\mu+\delta } \biggr)\bigl\langle I(t) \bigr\rangle -\frac{1}{2}\rho^{2} \biggl[- \biggl( \frac {\gamma+\mu}{\mu+\delta} \biggr)^{2}\bigl\langle I(t)\bigr\rangle ^{2} \biggr] \\ &{}+\frac{\rho}{t} \int_{0}^{t}S(r)\,dB(r) \\ =& \frac{\beta\varLambda}{\mu} \biggl[1-\frac{\mu ((\gamma +\mu)+\frac{1}{2}\rho^{2} (\frac{\varLambda}{\mu} )^{2} )}{\beta\varLambda} \biggr]- \biggl( \frac{\beta(\gamma+\mu )}{\mu+\delta} \biggr)\bigl\langle I(t)\bigr\rangle \\ &{}+2\frac{\varLambda}{\mu} \biggl(\frac{\gamma+\mu}{\mu+\delta } \biggr)\bigl\langle I(t) \bigr\rangle -\frac{1}{2}\rho^{2} \biggl[- \biggl( \frac {\gamma+\mu}{\mu+\delta} \biggr)^{2}\bigl\langle I(t)\bigr\rangle ^{2} \biggr] \\ &{}+\frac{\rho}{t} \int_{0}^{t}S(r)\,dB(r) \\ =& \frac{\beta\varLambda}{\mu} \biggl[1-\frac{1}{\tilde{R}} \biggr]- \biggl( \frac{\beta(\gamma+\mu)}{\mu+\delta} \biggr)\bigl\langle I(t)\bigr\rangle \\ &{}+2\frac{\varLambda}{\mu} \biggl(\frac{\gamma+\mu}{\mu+\delta } \biggr)\bigl\langle I(t) \bigr\rangle -\frac{1}{2}\rho^{2} \biggl(- \biggl( \frac {\gamma+\mu}{\mu+\delta} \biggr)^{2}\bigl\langle I(t)\bigr\rangle ^{2} \biggr) \\ &{}+\frac{\rho}{t} \int_{0}^{t}S(r)\,dB(r). \end{aligned}$$

If condition (2) is satisfied, then

21 $$\begin{aligned} \lim_{t\rightarrow\infty}\sup\frac{\log I(t)}{t}\leq\beta \frac {\varLambda}{\mu} \biggl(1-\frac{1}{\tilde{\Re}} \biggr)< 0, \end{aligned}$$

and conclusion (16) is proved. Next, according to inequality (19)

$$\begin{aligned} \frac{\log I(t)-\log I(0)}{t} \leq&\beta\bigl\langle S(t)\bigr\rangle -( \gamma+\mu)-\frac{1}{2}\rho ^{2}\bigl\langle S(t)\bigr\rangle ^{2}+\frac{\rho}{t} \int_{0}^{t}S(r)\,dB(r) \\ =&-\frac{1}{2}\rho^{2} \biggl(\bigl\langle S(t)\bigr\rangle -\frac{\beta}{\rho ^{2}} \biggr)+\frac{\beta}{2\rho^{2}}-(\gamma+\mu)+ \frac{\rho}{t} \int_{0}^{t}S(r)\,dB(r). \end{aligned}$$

If condition (1) is satisfied, then

22 $$\begin{aligned} \frac{\log I(t)}{t} \leq&\frac{\beta}{2\rho^{2}}-(\gamma+\mu)+ \frac{\rho}{t} \int _{0}^{t}S(r)\,dB(r)+\frac{\log I(0)}{t}, \end{aligned}$$

and conclusion (15) is proved. We have

$$\begin{aligned} \lim_{t\rightarrow\infty}\frac{\log I(t)}{t} \leq&-(\gamma+ \mu)+\frac{\beta}{2\rho^{2}}< 0 \quad\text{is a.s.} \end{aligned}$$

According to (15) and (16)

23 $$\begin{aligned} \lim_{t\rightarrow\infty} I(t)=0. \end{aligned}$$

Now, from third equation of system (1), it follows that

24 $$\begin{aligned} R(t)=e^{-(\mu+\delta)t} \biggl[R(0)+ \int_{0}^{t}\delta I(r)e^{(\mu+\delta)r}\,dr \biggr]. \end{aligned}$$

By applying the L’Hospital’s rule to the previous result, we have

25 $$\begin{aligned} \lim_{t\rightarrow\infty} R(t)=0. \end{aligned}$$

From Eq. (4), it follows that

$$\begin{aligned}& N(t)=e^{-\mu t} \biggl[N(0)+\frac{\varLambda}{\mu}e^{\mu t} \biggr], \\& S(t)+I(t)+R(t)=\frac{ [S(0)+I(0)+R(0)+\frac{\varLambda}{\mu }{e^{\mu t}} ]}{e^{\mu t}}, \\& \lim_{t\rightarrow\infty}S(t)=\lim_{t\rightarrow\infty} \biggl[ \frac{\{S(0)+I(0)+R(0)+\frac{\varLambda}{\mu}{e^{\mu t}}\}}{e^{\mu t}}-I(t)-R(t) \biggr], \\& \lim_{t\rightarrow\infty}S(t)=\frac{\varLambda}{\mu}. \end{aligned}$$

Hence, we have completed the proof. □

## Persistence

This section concerns the persistence of system (1).

### Theorem 5.1

*Suppose that* $\mu>\frac{\rho^{2}}{2}$. *Let* $(S(t),I(t),R(t))$*be any solution of model* (1) *with initial conditions* $(S(0),I(0),R(0))\in R_{+}^{3}$. *If* $\tilde{\Re}>1$, *then*

26 $$\begin{aligned}& \lim_{t\rightarrow\infty}\bigl\langle S(t)\bigr\rangle = \frac{\varLambda }{\mu}-\frac{1}{\mu} \biggl(\frac{(\gamma+\mu)(\mu+\delta )-\gamma\delta}{\mu+\delta} \biggr) \bigl\langle I(t)\bigr\rangle \end{aligned}$$

27 $$\begin{aligned}& \phantom{\lim_{t\rightarrow\infty}\bigl\langle S(t)\bigr\rangle }=\frac{\varLambda}{\mu}-\frac{\frac{\beta\varLambda}{\mu } [1-\frac{1}{\tilde{R}} ]}{ (\beta-\frac {2\varLambda}{\mu} )}, \end{aligned}$$

28 $$\begin{aligned}& \lim_{t\rightarrow\infty}\bigl\langle I(t)\bigr\rangle = \frac{\frac{\beta \varLambda}{\mu} [1-\frac{1}{\tilde{R}} ]}{ (\frac {\gamma+\mu}{\mu+\delta} ) (\beta-\frac{2\varLambda }{\mu} )}, \end{aligned}$$

29 $$\begin{aligned}& \lim_{t\rightarrow\infty}\bigl\langle R(t)\bigr\rangle = \frac{\gamma }{\gamma+\mu} \frac{\frac{\beta\varLambda}{\mu} [1-\frac {1}{\tilde{R}} ]}{ (\beta-\frac{2\varLambda}{\mu} )}. \end{aligned}$$

### Proof

We have

$$\begin{aligned} \frac{\log I(t))}{t} \leq& \frac{\beta\varLambda}{\mu} \biggl[1-\frac{1}{\tilde{R}} \biggr]- \biggl(\frac{\gamma+\mu}{\mu+\delta } \biggr) \biggl(\beta-\frac{2\varLambda}{\mu} \biggr)\bigl\langle I(t)\bigr\rangle \\ &{}+\frac{\rho}{t} \int_{0}^{t}S(r)\,dB(r)+\frac{\log I(0)}{t}. \end{aligned}$$

We apply the limit

$$\begin{aligned} \lim_{t\rightarrow\infty}\bigl\langle I(t)\bigr\rangle =& \frac{\frac{\beta \varLambda}{\mu} [1-\frac{1}{\tilde{R}} ]}{ (\frac {\gamma+\mu}{\mu+\delta} ) (\beta-\frac{2\varLambda }{\mu} )}. \end{aligned}$$

Using Eq. (17) we have

$$\begin{aligned} \lim_{t\rightarrow\infty}\bigl\langle S(t)\bigr\rangle =& \frac{\varLambda }{\mu}-\frac{1}{\mu} \biggl(\frac{(\gamma+\mu)(\mu+\delta )-\gamma\delta}{\mu+\delta} \biggr) \lim_{t\rightarrow\infty }\bigl\langle I(t)\bigr\rangle \\ =&\frac{\varLambda}{\mu}-\frac{\frac{\beta\varLambda}{\mu } [1-\frac{1}{\tilde{R}} ]}{ (\beta-\frac {2\varLambda}{\mu} )}. \end{aligned}$$

Furthermore,

$$\begin{aligned} \frac{R(t)-R(0)}{t} =& \gamma\bigl\langle I(t)\bigr\rangle -(\mu+\delta) \bigl\langle R(t)\bigr\rangle . \end{aligned}$$

By applying the limit $t\rightarrow\infty$, we have

$$\begin{aligned} \lim_{t\rightarrow\infty}\bigl\langle R(t)\bigr\rangle =& \frac{\gamma}{\mu +\delta} \lim_{t\rightarrow\infty}\bigl\langle I(t)\bigr\rangle \\ =& \frac{\gamma}{\mu+\delta} \frac{\frac{\beta\varLambda}{\mu } [1-\frac{1}{\tilde{R}} ]}{ (\frac{\gamma+\mu}{\mu +\delta} ) (\beta-\frac{2\varLambda}{\mu} )} \\ =& \frac{\gamma}{\gamma+\mu} \frac{\frac{\beta\varLambda}{\mu } [1-\frac{1}{\tilde{R}} ]}{ (\beta-\frac {2\varLambda}{\mu} )}. \end{aligned}$$

Hence, the proof is complete. □

## Numerical simulation

For the illustration of our obtained results, we use the values of the parameters and the variables given in Table 2.

**Table 2:** Values of variables and parameters for numerical solution

Variables and parameters	Values of variables and parameters
*S*(*t*)	59
*I*(*t*)	40
*R*(*t*)	30
*Λ*	0.008
*β*	0.002
*μ*	0.001
*γ*	0.02011
*ρ*	0.0045
*δ*	0.001

**Figure 1:**
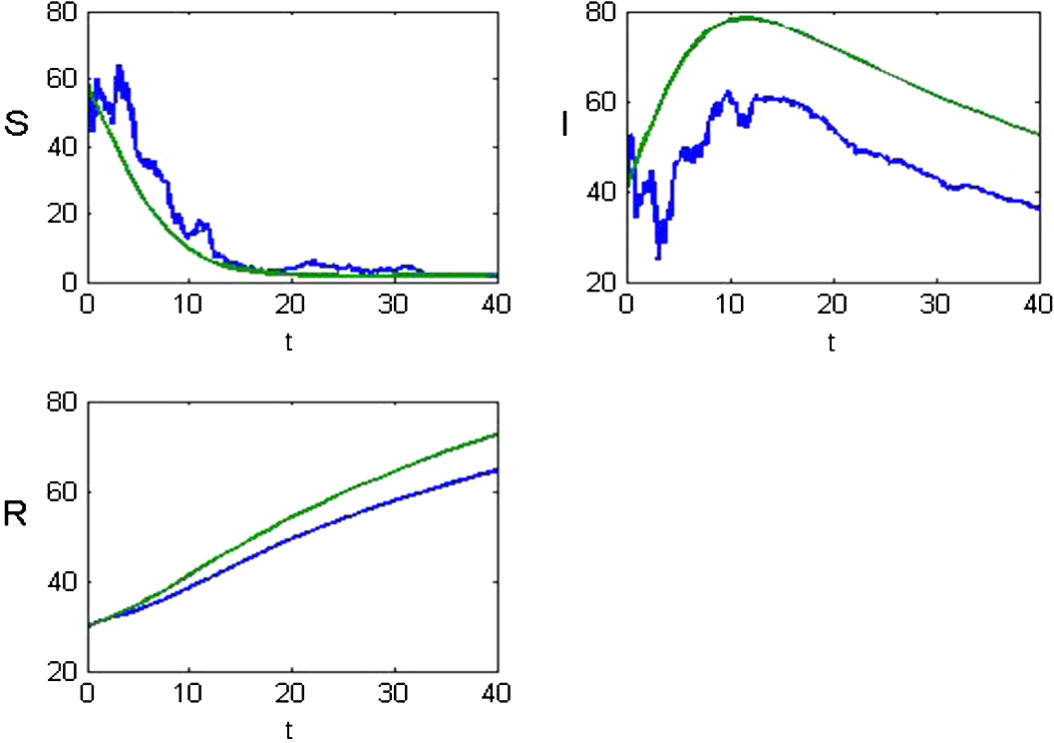
Graphs of (S) susceptible community using a deterministic method (green line) and from a stochastic solution (blue line), (I) infected people by coronavirus using a deterministic method (green line) and from a stochastic solution (blue line) and (R) recovered using a deterministic method (green line) and from a stochastic solution (blue line). The stability of stochastic graphs shows a better expression than deterministic graphs

Now for the numerical simulation, we use Milstein’s higher order method [40]. The results obtained through this method are shown graphically in Fig. 1 for both deterministic and stochastic forms.

## Conclusion

In this work, a formulation of a stochastic COVID-19 mathematical model is presented. The sufficient conditions are determined for extinction and persistence. Furthermore, we discussed the threshold of proposed stochastic model when there is small or large noise. Finally, we showed numerical simulations graphically with the help of software MATLAB. The conclusions obtained are that the spread of COVID-19 will be under control if $\tilde{R}<1$ and $\rho^{2}\leq \frac{\beta\mu}{\varLambda}$ means that white noise is not large and the value of $\tilde{R}>1$ will lead to the prevailing of COVID-19.
